# Effects of one hour daily outdoor access on lying and sleeping postures, and immune traits of tethered cows

**DOI:** 10.5713/ab.23.0011

**Published:** 2023-02-27

**Authors:** Huricha, Kyoko Horaguchi, Yuichiroh Shiiba, Sachi Tanaka, Ken-ichi Takeda

**Affiliations:** 1 Faculty of Agriculture, Shinshu University, Kamiina, Nagano 399-4598, Japan; 2 Graduate School of Science and Technology, Shinshu University, Kamiina, Nagano 399-4598, Japan; 3 Institute of Agriculture, Academic Assembly, Shinshu University, Kamiina, Nagano 399-459 Japan

**Keywords:** Animal Welfare, Dairy Cows, Immune Function, Lying, Outdoor Access, Tethering

## Abstract

**Objective:**

We investigated the effects of outdoor access for 1 h per day on the animal welfare (AW) of tethered cows, in terms of lying and sleeping postures, and immune function.

**Methods:**

A total of five dry cows were tethered all day indoors (tethering) for 30 days and then tethered indoors with 1 h daily outdoor access (ODA-1h) for 30 days. To analyze the effects of ODA-1h, we calculated the total duration and bout frequency per day, and bout duration of lying and sleeping postures during the last five days of each treatment period. We also analyzed the populations of T cells, B cells, and NK cells in peripheral blood mononuclear cells (PBMC) by fluorescence-activated cell sorting and determined the concanavalin A (Con A) -induced proliferation rate of T cells.

**Results:**

The mean total time per day of lying during the ODA-1h treatment was significantly shorter than that during the tethering treatment (p<0.001). The Con A-induced proliferation rate of T cells during the ODA-1h treatment was significantly higher than that during the tethering treatment (p = 0.007). The proportion of NK cells in PBMC during the ODA-1h treatment tended to be higher than that during the tethering treatment (p = 0.062).

**Conclusion:**

Although ODA-1h may decrease lying time, it increases the available space for tethered cows towards that typically found in grazing and free barn feeding systems. This increased available space promotes the expression of normal behaviors such as walking and social behaviors except lying and may also improve the immune function of tethered dry cows, thereby improving their overall welfare.

## INTRODUCTION

The tethering system can reduce the animal welfare (AW) of dairy cows in various ways, such as reducing normal behavior expression [1], increasing abnormal behavior expression [2], and worsening health status [3]. Therefore, it is important to introduce measures to achieve normal behavior and improve the health status of dairy cows in tethering systems rather than relying on continuous tethering. From an AW perspective, it is prohibited to tether cows indoors for a long time and regular outside access is required in some countries [4,5]. However, many dairy farms worldwide still adopt a tethering system, for example, 74% in Canada, 39% in the United States, and 82% in Austria [6]. In Japan, approximately 70% of dairy farms still adopt a tethering system [7]. Therefore, the AW of dairy cows is a major problem under the increased land restrictions in Japan.

Outdoor access is one improvement measure that is reported to increase AW levels in tethered cows [8]. However, further research is needed on the time and frequency of outdoor access and its merits for raising AW at the production sites of tethered cows. Access to an exercise yard for 1 h per day is sufficient for cows to exhibit normal levels of locomotor activity and other behaviors [9], and to reduce abnormal behaviors [1,9]. However, the effect of outdoor access 1 h per day on lying, one of the AW criteria, is not yet clear [6]. Previous studies have shown that outdoor access reduces the incidence of foot health problems and parturition-related problems in tethered cows [10], as well as improving their immune function [11]. The methods used in these studies involved forcing the cows to exercise. This forced exercise, albeit at a low speed, may cause stress to the cow and may also create a labor burden on the farmer. In addition, there are no studies on the effects of 1 h daily outdoor access on immune function in dairy cows. In this study, we evaluated the effects of 1 h daily outdoor access on AW in terms of the immune function and lying and sleeping postures of tethered cows.

## MATERIALS AND METHODS

This study was performed in accordance with the Animal Experimental Regulations of Shinshu University (Approval No. 020031).

### Animals

We analyzed 5 non-pregnant, non-lactating Holstein cows reared on a farm in the Education and Research Center of Alpine Field Science, Faculty of Agriculture, Shinshu University (Table 1).

### Breeding facility and management

**Figure 1:**
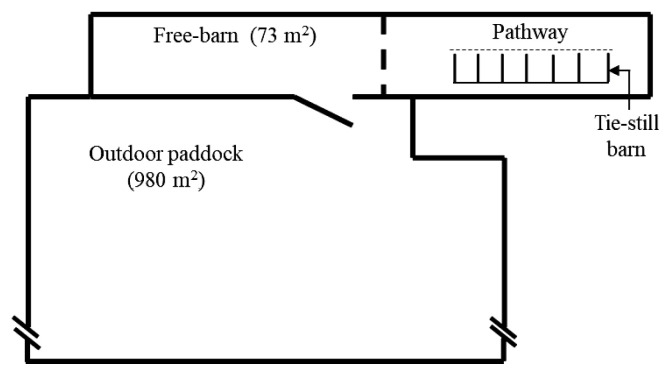
Structure of breeding facility.

**Figure 2:**
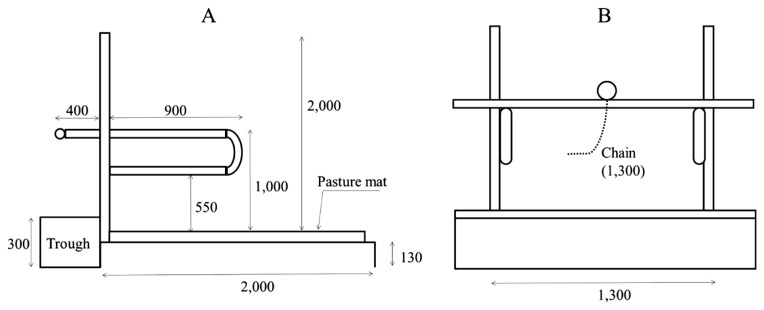
Tie-stall structure and dimensions (side, A; front, B; unit, mm).

The breeding facility consisted of a tie-rail-type stall (known as the New York tie stall), an indoor free barn, and an outdoor paddock (Figure 1). The detailed construction of the tie-rail-type stall is illustrated in Figure 2. During the experiment, there was no grass to eat in the outdoor paddock.

Under feeding management, 2 kg of hay cubes and 12 kg of oat hay were divided into equal amounts per cow, and fed at 8:30 h and 16:00 h at tie-rail-type stalls. The cows could ingest mineral salts and water freely. Prior to the experiment, all cows were usually kept in a tie-rail-type stall during feeding and at night, and in a free barn and an outdoor paddock so that they could move freely during the daytime.

### Experimental design

Two different treatments were applied during the experimental period: 30 days of all-day tethering (tethering), followed by 30 days of tethering with outdoor access for 1 h continuously (approx. 9:30 to 10:30 h) during the day (ODA-1h). Higashiyama et al [12] reported that when cattle were changed from grazing to indoor tethering, urinary cortisol levels were higher in the first week and remained the same level as during grazing from the second week. This finding suggests that the response of cattle to environmental changes may become weaker from the second week. Therefore, the treatment period in this study was set as 30 days over 2 weeks, respectively. During tethering, cows were kept in a tie-rail stall barn (Figure 2). During the ODA-1h treatment, cows were tethered except for a 1 h period after morning feeding, when the cows could move freely in the tie-rail stalls, free barns, and outdoor paddock. Lameness, infection, or other health problems were not observed during the experiment.

### Behavioral observations

Behavioral observations were made during the last 5 days of each treatment. Surveillance cameras were used to record the behaviors of the cows. The observation items were lying, and rapid eye movement (REM) sleeping posture in a lying position. REM sleeping posture is defined as when the cow does not move its head to contact the ground, or when its head is turned backward to touch its own body [13]. Behavioral data were first collected for the total duration and bout frequency per day (24 h) and mean bout duration in lying or sleeping postures, and then calculated as an average for each cow during the last 5 days of each treatment. Walking steps and social behaviors were observed during 1 h outdoor access on the last 5 days of ODA-1h.

A temperature–humidity index (THI) calculated from the temperature and humidity was introduced into the analysis to eliminate the influence of heat stress on each behavior. A data logger (TR-72Ui; T&D Co. Ltd., Matsumoto, Japan) was used to measure and record temperature and humidity data at intervals of 30 minutes from the beginning to the end of the experiment. THI was calculated as THI = (DB×0.8 + [RH×0.01]×[DB − 14.4])+46.4 [14], where DB is the dry bulb temperature and RH is the relative humidity. In this study, THI ranged from 64.40 to 70.89 during the tethering treatment and from 61.24 to 68.47 during the ODA-1h treatment.

### Immune function

A 30 mL blood sample was collected from the jugular vein of each cow during the morning feeding time on the last day of each treatment period. Samples were taken using a blood collection tube containing heparin sodium (Terumo, Tokyo, Japan). Peripheral blood mononuclear cells (PBMC) were isolated using the method of Tanaka et al [15]. Blood was diluted 1:1 with phosphate-buffered saline (PBS) and then layered on Lympholyte-H (Cedarlane, Ontario, Canada). After centrifugation at 600×g for 30 min at 4°C, the buffy coat layer of PBMC was collected, washed in PBS, and hypotonically lysed with 0.83% ammonium chloride in Tris-HCl buffer. After washing PBMC with RPMI-1640 medium with 10% fetal bovine serum and adjusting the cell count of PBMC to 1×10^7^ cells/mL, and the populations of T cells, B cells, and NK cells in PBMC were analyzed by flow cytometry. We also analyzed the concanavalin A (Con A)-induced proliferation rate (lymphocyte blastogenesis) of T cells.

The monoclonal antibodies (mAbs) MM1A, CC21, and AKS1 (Bio-Rad Laboratories, Inc., Tokyo, Japan) were used for flow cytometry. T cells were detected using MM1A. B cells were detected using CC21. NK cells were detected using AKS1. CC21 and AKS1 were diluted 1:50 with PBS, and MM1A was diluted 1:10. PerCP-labeled rat anti-mouse IgG1 (Becton Dickinson, San Jose, CA, USA) was used as the secondary antibody, specifically labeled mAbs.

Flow cytometry was performed as described by Tanaka et al [15]. PBMC samples of 1×106 cells were incubated with each primary mAb for 15 min at 4°C, washed once, and resuspended in PBS. The cells were incubated with secondary antibody for 15 min at 4°C, washed, and analyzed using a FACS Calibur system (Becton Dickinson, USA). All data acquired by FACS Calibur were analyzed using FlowJo (Treestar, Ashland, OR, USA). FACS Calibur was used to obtain optical signatures of 50,000 cells per sample. The control samples from each test sample were stained with only the secondary antibody. The percentage of fluorescent positives was recorded using a FACS Calibur.

The Con A-induced proliferation rate of T cells was determined as described by Yamamoto et al [16]. T cell proliferation was analyzed using the Cell Counting Kit-8 (CCK-8; Dojindo Laboratories, Tokyo, Japan). PBMC samples of 5×105 cells were seeded in 96-well flat-bottomed plates, stimulated with 1 μg/mL Con A, and incubated at 37°C for 48 h. Non-stimulated samples were incubated at 37°C for 48 h with RPMI-1640 medium instead of Con A. Subsequently, 100 μL of CCK-8 solution was added to each well, and the samples were incubated at 37°C for a further 2 h. The absorbance was determined at 450 nm using a microplate reader (Multiskan Sky; Thermo Fisher Scientific, Tokyo, Japan).

### Statistical analysis

The effect of ODA-1h on each cow behavior was estimated using the linear mixed model function “lmer” of R version 4.1.0 [17]. Specifically, the behavioral data in the two treatments were taken as the target variable, tethering was set to 0, and ODA-1h was set to 1 as the explanatory variable. Individuals were treated as a random effect. In addition, THI was introduced into the model as an explanatory variable.

The effect of ODA-1h on the immune function of cows was estimated using the paired t-test in R version 4.1.0 [17].

## RESULTS AND DISCUSSION

During 1 h outdoor access of ODA-1h treatment in this study, the cows used the outdoor environment, except on rainy days. The mean total duration and bout frequency per day, and mean bout duration of lying during the tethering and ODA-1h treatments are shown in Table 2. The mean total duration per day of lying in ODA-1h was significantly shorter than that in tethering (regression coefficient = −1.68, p<0.001; Table 2). The number of walking steps per 1 h outdoor access was 323.7±69.8 (mean±standard error [SE]). The frequency of social behaviors per 1 h outdoor access of 5 cows was 5.6±4.0 (mean±standard deviation [SD]). These behaviors were not observed during the same tethering period as the 1 h outdoor access of ODA-1h.

Lying is a high-priority behavior of cows [18], which can reflect their AW levels [19]. Some AW grade evaluation criteria recommend measuring the lying time of cows [4,5]. The daily lying time of dairy cows varies greatly depending on the type of breeding system, for example, 12.5 h for tethering, 10.6 h for free stall, and 9.5 h for grazing [20]. This suggests that the differences in lying time reflect the differences in the behavioral restrictions of the cows. In general, the lying time in free barns is shorter than that in tethering [20]. In addition, grazing systems are perceived to offer greater behavioral freedom than continuously housed systems [21]. In tethering systems, in addition to restrictions on walking, there are many abnormal behaviors [6]. In this study, the mean total duration and bout frequency per day of lying during the ODA-1h treatment were less than those during the tethering treatment, but the normal behaviors such as walking and social behaviors increased. In other words, ODA-1h may have promoted normal behavior expression except for lying, owing to the increased available space for tethered cows gained through outdoor access for 1 hour, towards that typically found in grazing or free barn feeding systems.

The lying posture of cows is affected by factors such as the quality of the floor mat [22] and health status [23]. The cow floor mat material used in this study was pasture mat (CORNES AG, Hokkaido, Japan). The pasture mat greatly reduces the pressure in the anterior knees of cows compared to previous rubber mats [24]. In addition, none of the cows in this study had any health problems, such as lameness or infectious diseases. However, the mean total duration per day and the mean bout duration of lying in the two treatments in this study were higher than those in tethered milking cows (mean daily lying time, 12.50 h; mean bout duration of lying, 1.14 h) [20]. This may have been related to amount of milk production. The studies have reported that daily lying time is negatively correlated with the amount of milk [25]. Since the cows used in this study were dry, it is possible that they had a longer daily lying time and bout duration than milking cows.

The mean total duration and bout frequency per day, and the mean bout duration of REM sleeping posture during the tethering and ODA-1h treatments are shown in Table 2. The mean total duration per day of REM sleeping posture during the ODA-1h treatment was significantly shorter than that during the tethering treatment (regression coefficient = −0.22, p<0.05; Table 2). This may be because the lying time was shorter during the ODA-1h treatment than that during the tethering treatment. In other words, because REM sleeping posture occurs in the lying posture [20], REM sleeping posture time may decrease with a decrease in lying time. There was no difference in the ratio of the total duration per day of REM sleeping posture to the total duration per day of lying between the two treatments in this study, which suggests that the ODA-1h treatment had no effect on REM sleeping posture. This finding is contrary to those of previous studies where animals spent more time in REM sleeping posture when AW was improved by improving the feeding environment [26]. The reasons for this difference require further investigation.

The average values of the immune characteristics during the tethering and ODA-1h treatments are shown in Table 3. The proportion of T cells during ODA-1h tended to be lower than that during tethering (t = 2.71, p = 0.053; Table 3). The Con A-induced proliferation rate of T cells during ODA-1h was significantly higher than that during tethering (t = −5.15, p = 0.007; Table 3). The proportion of NK cells during ODA-1h tended to be higher than that during tethering (t = 2.57, p = 0.0062; Table 3).

Hematological characteristics are the main indicators of animals’ environmental adaptation and, thus, their welfare [27]. Differences in feeding management can affect the hematological characteristics of cows [28]. For example, it has been reported that when housed cows are provided with an outdoor environment, their white blood cell population increases, suggesting increased immune system activity [28]. In this study, we also found that the Con A-induced proliferation rate of T cells of cows during ODA-1h was significantly higher than that during tethering, and the proportion of NK cells of cows during ODA-1h tended to be higher than that during tethering. The Con A-induced proliferation rate of T cells and increase in NK cells may be evidence of inflammation and other pathologies. However, in this study, no disease was found in the cows during the experimental period. In addition, the proportion of T cells during the ODA-1h treatment tended to be lower than that during the tethering treatment, indicating that there was no pathological evidence. One reason for the improved immune function of cows during ODA-1h may be their ability to walk. Research has suggested that, in humans, walking exercise activates NK cells and enhances lymphocyte blastogenesis [29]. In dry cows, the use of walking exercise is also thought to cause temporary increases in the proportion of T cells and in many T cell subsets, which affects the immune function of cows [11]. Outdoor sunbathing may also be related to the improved immune function in cows during ODA-1h. In breeding cows, sunbathing during a certain period in autumn was thought to hyperactivate immune function compared with that in the control group without sunbathing [30]. However, the mechanism underlying the effects of sunbathing or walking exercise on the immune function of cows remains unclear, and further investigation is required.

Our results suggest that, compared to tethering continuously, outdoor access for 1 h daily may decrease the lying time, but increase the available space of tethered dry cows. The increased available space promoted the expression of walking and social behaviors, except for lying, and may also improve the immune function of dry cows. Our study data and findings may help to promote AW for tethered dry cows. However, for milking cows, it is suggested that extreme walking can decrease their milk yield and change their milk composition [31]. These changes are due to the lack of additional supplements intake by milking cows to compensate for the increased energy requirements during walking [31]. In future research, the effect of 1 h daily outdoor access rather than extreme exercise on milk yield and milk composition of milking cows, the optimum size of outdoor paddocks for access and the difference between indoor and outdoor release sites will need to be clarified.

## Figures and Tables

**Table 1:** Basic details of the cows used in this study

Cow No.	8,219	7,637	7,649	7,802	7,834
Age	4	7	7	4	4
Weight (kg)	754	818	894	750	950

**Table 2:** Effect of outdoor access for 1 h per day (ODA-1h) on total duration and bout frequency per day and mean bout duration of each behavior for tethered dry cows

Behavior	Tethering (Mean±SE)	ODA-1h (Mean±SE)	ODA-1h regression coefficient	p-value
Lying				
Total duration (h/d)	14.44±0.94	13.12±1.01	−1.68	<0.001
Bout frequency (bouts/d)	10.12±2.12	8.84±0.83	–1.62	0.077
Mean bout duration (h)	1.50±0.42	1.52±0.42	0.05	0.683
REM sleeping posture				
Total duration (h/d)	1.51±0.47	1.40±0.50	−0.22	0.047
Bout frequency (bouts/d)	18.16±7.98	18.60±10.29	−1.19	0.542
Mean bout duration (h)	0.09±0.02	0.08±0.02	−0.01	0.088
REM sleeping time/lying time (%)	10.68±4.07	10.81±4.55	−0.46	0.561

SE, standard error; REM, rapid eye movement.

**Table 3:** Effect of outdoor access for 1 h per day (ODA-1h) on various immune traits for tethered dry cows

Immune trait	Tethering (Mean±SD)	ODA-1h (Mean±SD)	t	df	p-value
T cell (%)	38.56±5.40	30.95±5.95	2.71	4	0.053
B cell (%)	18.99±4.38	21.41±5.85	−0.74	4	0.499
NK cell (%)	2.75±0.70	4.07±1.11	−2.57	4	0.062
Con A-induced proliferation rate of T cells	4.35±0.82	8.14±1.18	−5.15	4	0.007

SD, standard deviation.
